# Solidarity and its decoloniality in global health ethics

**DOI:** 10.1186/s12939-025-02380-y

**Published:** 2025-01-16

**Authors:** Ademola Kazeem Fayemi, David Gerrard Kirchhoffer, Bridget Pratt

**Affiliations:** 1 Queensland Bioethics Centre, Australian Catholic University, Brisbane, Australia; 2 School of Population and Global Health, University of Melbourne, Melbourne, Australia

## Abstract

Solidarity is one of the emerging values in global health ethics, and a few pieces of bioethics literature link it to decoloniality. However, conceptions of solidarity in global health ethics are influenced primarily by Western perspectives, thus suggesting the decolonial needs to include non-Western perspectives. This article explores a decolonial interpretation of solidarity to enrich our understanding of solidarity. It employs a palaver approach, typical of African (Yorùbá) relational culture, in developing a conception of solidarity grounded in a beehive metaphor. Through a decolonial methodological approach, this article posits that a beehive metaphor allegorically symbolises solidarity. In this decolonial interpretive account, solidarity embeds relational virtues and duties that foster harmony. Solidarity is a positively oriented affective disposition with people with whom one shares similar circumstances for harmonious well-being through concerted efforts. This article addresses five potential objections to this account of solidarity in global health ethics and consequently explores what an African account of solidarity means for global health research funding. This article concludes that the palaver decolonial approach from the Global South has implications for expanding conceptual perspectives on solidarity in global health ethics.

## Introduction

Global health ethics involves the analysis of moral issues and the application of morally acceptable solutions to health issues emanating from “public health, healthcare, and health research in a global or global South context” [1, 2]. This application entails critically examining the values and principles used in analysing moral issues identified in those contexts. Solidarity has long been one of the core values identified in guiding global health ethics [3–6]. Although its meaning, nature, and boundaries are still being contested, the emerging consensus is that solidarity holds some promising implications for addressing moral issues requiring global action. Shapiro and Benatar [3] noted that “some degree of solidarity … is not an excessive moral requirement” for equitable global health, whereas Benatar and Peter Singer [5] maintained solidarity as the “most important value underpinning” global health. Its importance was highlighted during the COVID-19 pandemic, where the failure of the global community to internalise the ideal of solidarity resulted in inequities in access to medicine, specifically in vaccine distribution [7–9].

Recent scholarship emphasises the necessity of solidarity and health equity as values in global health ethics [10–15]. Many of these works on solidarity, which have used standard methods in bioethics, are applications of Western conceptions of solidarity to global health, global health governance, community engagement, and research regulations in global health. With an underexploration of solidarity conceptions from the Global South,^1^ there is thus a need to decolonise the concept of solidarity in global health ethics by including non-Western perspectives. Relying on Western methods and applying mainly Western concepts is part of a broader problematic trend of epistemic injustice in global health ethics and bioethics. The resultant calls to address epistemic injustice and missing voices from the global South in global health ethics necessitate exploring and bringing to focus African views on solidarity and its decoloniality. Decoloniality is an evolving movement challenging Western epistemic hegemony, cultural imperialism, and power inequities historically created by colonialism [17]. “Rooted in Indigenous thought and practice about nature, community, and solidarity,” [18] decoloniality is an alternative mode of thinking to nation-statism, colonialism and coloniality with their enabled modes of knowing. Global health ethics involves, among other complex processes, sustained critical reflections on identifying, desilencing, and disentangling values and norms from the Global South that can be serviceable in rectifying the undue influences of historical colonialism, thus inhibiting the advancement of global health equity and justice.

This article explores a decolonial conceptualisation of solidarity in global health ethics. The justification for articulating an African perspective on solidarity, as opposed to other less-heard voices from the Global South, is to provide a starting point for a conversation with normative ideas from non-Western and Western epistemologies. By articulating a conception of solidarity from a marginal cultural lens, we may mainstream a more epistemically inclusive conception of solidarity in global health ethics.

While there are a few works on the African moral conception of solidarity applied to global health issues, they do not specifically focus on decolonising the approach used in arriving at African conceptions of solidarity [19–22]. What distinguishes the concept of solidarity proposed in this article is the methodology adopted, which draws on African ways of knowing. Decoloniality is advanced not only in the conceptual sense but also in a methodological sense by using a metaphoric approach to derive an African conception of solidarity. Indigenous knowledge in many African societies south of the Sahara is codified, transmitted and preserved across generations in metaphorical narratives such as tales, idioms, riddles and proverbs. In discovering the authenticity of African views on solidarity, metaphors and proverbs offer valuable literature, such as indigenous philosophy, and are sources of material for extending philosophical ideas about solidarity. This is especially the case in African culture and elsewhere, where orality constitutes a different way of expressing philosophy [23].

By adopting such a methodological approach, this article demonstrates how approaches from the Global South can generate ideas on solidarity in global health ethics. It, therefore, contributes to helping decolonise the concept of solidarity in global health ethics through a specific interpretation of a beehive metaphor. The use of the beehive is allegorical and not literal. This paper does not claim that the beehive is the perfect analogy, and the anthropomorphisation of animals to tell moral stories is very common in African folklore. Drawing on the Yorùbá example of an allegorical understanding of a beehive for thinking ethically, which is characteristic of oral traditions that use stories and metaphors to communicate ethical values and principles, this article aims to show that alternative ethical approaches from the global South can help think about and generate conceptions of solidarity.

In making “knowledge from the global South visible and valued in global health ethics,” Pratt and de Vries [1] called for the need to have zones of dialogues “between those based in the global North and South about the field’s underlying methods” and moral concepts. In response, this article seeks to initiate such dialogue by challenging the subordination and erasure of methodology from the Global South to conceptualise ethical values. Its employment of a metaphoric approach to knowledge production in global health ethics is thus intended as an instance of methodological decoloniality. As decolonial approaches in global health ethics and decolonial perspectives on solidarity from the Global South grow and are applied to topical issues in global health research discourses, “constellations of knowledge informed by different ways of understanding solidarity” might reasonably be envisioned [1].

In four parts, this article begins by introducing a metaphoric approach and using it to generate an African-inspired account of solidarity. It then highlights five possible objections against the proposed account of solidarity. Next, it provides an example of how such an account might be applied in the context of rethinking global health research funding. To conclude, this article identifies areas of future research on the value of solidarity in global health ethics.

## Beehive metaphor and an african conception of solidarity

This section draws on proverbs, oratures, and metaphors in Yorùbá^2^ culture to derive an African-inspired account of solidarity. A beehive metaphor is presented together with some selected proverbs that metaphorically describe acts and reality. The choice of the beehive metaphor is consciously informed by its social representation of power hierarchies that imaginatively mirror human society. While the metaphor is commonplace in Yorùbá thought, and in many African cultures, it is not unique to those contexts.

The transmission of embedded knowledge in oral tradition consists of passing on extant traditional ideas and having hermeneutic conversations and critical engagement with traditional ideas for contemporary relevance and situatedness [24, 25]. This approach is adopted in this article and simulates what is popularly called ‘palaver’ in many African cultures. The African palaver is a way of wittily participating, dialoguing, and building consensus among people and the community on any issue through the use of allegories and proverbs that identify problems, interpret their meanings, and suggest corrective guidelines [26–28]. Palaver is both “the art of conversation” and “a depolarising space for listening and engaging in open, collaborative, participatory dialogue, and mutual learning from one another” [28]. As a common tool in decision-making on everyday challenges, conflict resolution, and moral discernment in customary African societies, the palaver uses allegories, proverbs, fables or/and metaphors in explicating and mediating conversations to obtain points across. In the context of ethical values, the palaver is a methodology and a creative space for arriving at moral judgment and consensus. This paper introduces a palaver with extant conceptions of solidarity and other voices from the Global South. Yours is a contribution to that broader palaver. In contributing to this broader palaver and in line with the palaver model of not imposing moral precepts and actions but collectively discovering what kind of character is ideal, this article presents traditional solidarity through a beehive metaphor. Furthermore, it complements the virtues figuratively expressed in the beehive with some illustrative proverbs.

“The beehive is a space where bees distribute workforce among the hive’s needs, collect nutrients, regulate temperature, select location to colonise” [29]. It is an interactive space and structure of sociality where bees live and show their collective commitment to the hive’s survival. The beehive has many layers or cell structures with different stratifications of functions and activities, including foraging, construction, care for the young, and defence of the colony. From the queen, drones, to workers, there are divisions of labour in the activities and roles of bees in the hive. While some forage, clean, build, guard, make wax cells, fan watery nectar, or provide brood care for larvae and pupae, others either lay eggs or mate. The main role of the queen in the colony is to lay eggs; drones mate with virgin queens; and worker bees have the task of protecting the colony and caring for the drones, the queen, the larvae and the pupae.

While the drones and the queen have shared reproductive roles, they share a bond with the workers through their genotypic similarity. The queen bee is larger in size and stings. Although capable of stinging, the sting is not usually used in punishment or oppression. Through nonsynchronous waggle dances and stop signals, worker bees symbolically alert the hive to local environmental danger, look after one another, and communicate by pointing in the directions of nectar and water [30]. An important task of worker bees is the collection of nectar and pollen used in the production of honey, which is a primary source of energy in the hive. These various activities and role performances of bees show solidarity. The motivation for bees’ solidarity is the survival of the colony. For this reason, drones temporarily stay in the colony as they fly off in search of potential new queens to mate. While outside the colony, the drones do not last long. When the size of the hive becomes large, swarming occurs, and the adult queen bee leaves with some workers to establish a new colony.

The above social structure and behaviour of bees in a hive serve metaphoric purposes for further reflection on what solidarity entails. As a functional unit of cohesion, some symbolic virtues can be drawn from the instinctual behaviour of bees and the hive itself, conjunctively understood as beehive, henceforth. The beehive is a quintessential representation of integrated virtues, including humility, sharing, hospitality, cooperation, and participation. Each is necessary but not independently sufficient for solidarity. Allegorically, the beehive is a metaphor for solidarity, and it highlights the importance of cooperation, sharing, participation, hospitality, and humility in achieving harmonious living for the benefit of the community. Although the word ‘solidarity’ is literally translated in the Yorùbá language as ‘*ajowapo’* (“we exist together”), its metaphorical nuances go beyond the descriptive fact of collective existence. For Yorùbá, solidarity is a positively oriented disposition or norm prescribing virtues that lead to harmony towards the self and the ‘other’. Solidarity is thus understood as both in-group and out-group.

The beehive metaphor could be used to illustrate in-group solidarity within the bee colony itself. In-group solidarity means identifying and sharing with people who share similar categories (such as ethnicity, religion, gender, and nation). It seeks to achieve the yearnings of in-group members, promote the good of other group members, and strive towards internal social cohesion. Bees seem to work together cooperatively, demonstrating how members of the same group or community can collaborate for the collective good. On the surface, the beehive seems to emphasise in-group solidarity, and one may think, along with Selin Kesebir [29], that bees do not have out-group solidarity because they do not have flexibility and multiple identities that characterise humans; they are simply “superorganisms”. Out-group solidarity means identifying and sharing with people outside one’s immediate group. It seeks to achieve mutually beneficial goals involving the good of the ‘other’ excluded from the in-group’s shared interests and striving toward external social cohesion.

However, a deeper heuristic analysis will show that the beehive metaphorically allows an internal dialogue and solidaristic connection with the outgroup. Indeed, bees (drones) interact with other bee colonies for purposes such as mating and facilitating species continuation, and some bee species even engage in mutual relationships with other species within biological families when foraging and pollinating. For example, consider male solitary bees and their search for female honeybees to mate. This study revealed that the behaviours and activities of bees are not always exclusive to their in-group circles; out-group solidarity with bees outside biological groupings also occurs. Despite the potential polarity of in-group and out-group bees, there is a common interest in the collection of pollen and nectar for the survival and pollination of flowers, which, in turn, leads to the production of fruits or seeds. Arguably, the metaphor does not need to be limited to a specific community of in-group(s); it is compatible with the idea of out-group solidarity.

Like solitary and honeybees, which belong to the same biological family of bees despite their group variation, some deductions can be made concerning humans, who are also capable of being within and between solidarity groups. If the solitary and honey bees are interpreted as in/out-groups to whom we owe social goods within the human species and the hive as a metaphor for the global space, then the layers of solidarity (in-group and out-group) become fluid. Everybody is part of the world, whether within the in-group or out-group category. Indeed, a Yorùbá proverb that mirrors the fluidity of solidarity identification and belongingness is *“aaye daabi afara oyin*,* ona sooro; yaara ototo*” (“The world is a beehive with similar entrance but living in different cells”). This proverb speaks to the hive analogously representing the world that houses interrelated, interconnected, and interdependent beings but sleeping in different rooms (spaces and other identities) that broadly divide them into forming in-group and out-group solidarities. The hive is structured in different cells and layers of bees, and each bee depends on one another for collective survival. If the bees are taken as human species, the beehive metaphor shows that everybody can be part of the beehive, regardless of the cell one inhabits and dwells within the hive. Therefore, the hive descriptively shows the world as an interconnected and interdependent space.

**Fig. 1:**
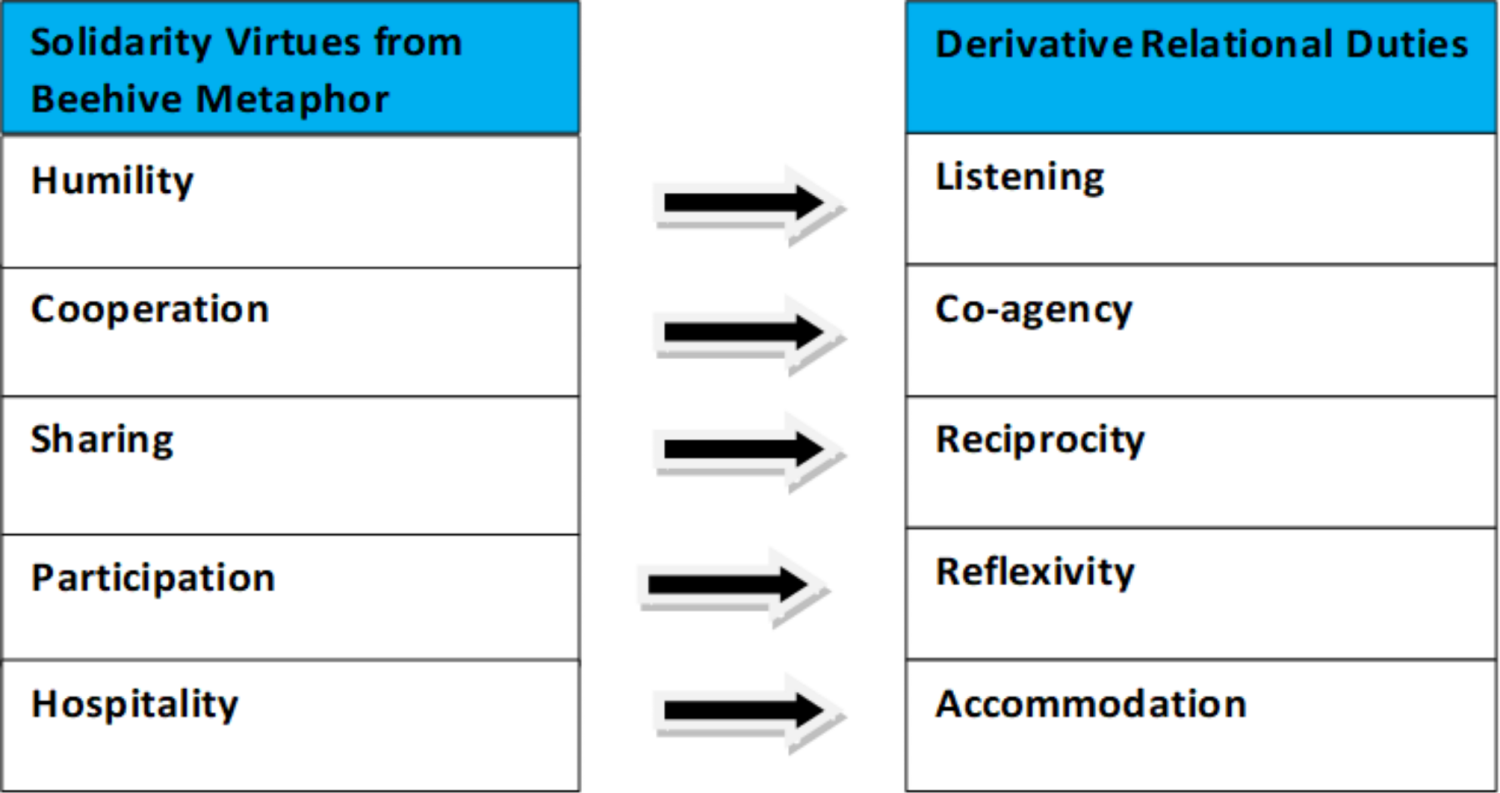
Solidarity virtues and corresponding relational duties

However, it also normatively embeds virtuous ideals that make the space unchaotic by promoting an interplay of responsibilities and duties among its constituents. The membership of an in-group does not limit responsibilities and duties to only people within that group. Whether at the in-group or out-group level, the *relational virtues* of humility, sharing, cooperation, participation, and hospitality are essential and derived from Yorùbá probers. They further imply *corresponding duties* of listening, coagency, reciprocity, reflexivity, and accommodation (see Fig. 1). The duty is understood here as a sense of responsibility in actioning relational virtues. Becoming virtuous requires showing isolated or single virtues and observing good character through consistently displaying the virtues and upholding their corresponding duties. Virtues entail duties because “the carrying out of a person’s moral duty depends on character” [31]. Observing moral obligations, accepting responsibilities, and guiding actions in specific ways are functions of an individual’s character quality.

### Virtues and duties of solidarity

The behaviours of bees in the hive reveal the virtue of humility. When bees work together, they do so without seeking recognition or personal gain. The different kinds of bees in the hive, whether drones, workers or queens, act on their roles without showing self-arrogance. Although bees are naturally instinctive, their social organisation and activities, as well as their seeming metaphoric demonstration of virtues of humility and cooperation, cumulatively lead to the production of honey in the hive. Humility is “a disposition not to think too much of oneself” [32]. To be a humble moral agent, one needs to be “unassuming, relatively unconcerned that her status be greater than others and not wanting to impose on others without giving their interests at least due consideration” [32]. Humility, for philosopher Thaddeus Metz, conjunctively consists of making reasonable demands on others, having a proportionate value of oneself, and acting selflessly [32]. It is essentially about “having respect for others’ points of view” and “it is crucial to developing others regarding virtues and facilitating “human collaborative relationships” [33]. The arrogance of a few or many ruins the capacity for solidarity in human relationships. Respecting opinions, regardless of the social stratification of opinions, is a harbinger of cooperation. A cogent Yorùbá proverb that depicts humility as a concomitant virtue to success is (i)^3^ “*Suru baba iwa; iwapele oba awure*” [34] (literally translated, this proverb means patience is a primary virtue; humility is the greatest gateway to good fortune). Where there is failure of humility, power struggle for arrogant dominance festers with disgruntlement within the network. However, where humility is extolled as a virtue, members of solidaristic groups tend to share the burden and cooperate with one another within and outside their groups.

An inferential duty from the virtue of humility is, therefore, listening. Members of an in-group must listen to one another. Humility is capable of refinement when actors listen more to the silenced and ignored voices of others (marginalised groups within the in-/outgroups), regardless of their status and positionalities. The notion of “‘voice’ draws attention to the lived experience of the other; it is situated, engaged and relational, launching inroad to the lived experience of the other [marginalised groups]” [32]. Humility creates opportunities for dialogues with marginalised groups hitherto not recognised within social arrangements. Solidarity with others is difficult, if not impossible, when people do not respect the duty of listening to others [group members]; it is through listening that trustworthy relationships are built within (and among) the in-groups and out-groups. “A relationship of solidarity characteristically is one in which a person feels a certain way about another consequent to attentive awareness of him” [35]. This is done by listening to the other and reflecting on what the other “might be holding back or unable to express” [35].

An instructive Yorùbá proverb on humility states (ii) ‘*Mo gbon tán*,* mo moràn tán*,*’ kì í je kí agbon lóró bí oyin.* (‘I am allwise, I am all-knowing,’ keeping the wasp from having as much venom as the bee) [36]. This proverb states that the bee instinctively listens to instructions in the hive on how to infuse its sting with venom. Nevertheless, the wasp is arrogant, thinking it knows everything. The salient point underscored by this proverb is that “to learn, you must be willing to listen” [37].

The beehive is a site of cooperation. The structure, as well as the survival of the colony, is a function of cooperation among bees. In terms of foraging, defense of the colony against predators, caring for the house bees, or adapting to environmental conditions, there is cooperation among the bees. Cooperation is one of the virtues of solidarity, which means working together with the ‘other’ to enable a better life and achieve positively oriented goals. Cooperating with others in the network of people with similar and relevant situations is fundamental in solidaristic relationships. Cooperation presupposes the identification of similar conditions or circumstances; it allows humans to think in ‘we-terms.’ Bees identify with one another as belonging to the species of social insects. This identification of ‘we-ness’ brings about respect for each bee, regardless of how infinitesimal it is in the beehive. Identifying with respect to one another allows cooperation. A Yorùbá proverb that aptly captures cooperation states that (iii) *“*Ọ*tún w*ẹ *òsì*,* òsì w*ẹ *otún l*ọ*wo fi *ḿ*mo*” (Hands becomes clean when the right hand washes the left hand and the left hand washes the right hand) [36]. This proverbial lore calls attention to the importance of cooperation within the in-group and between out-group members in making for harmony, protecting the group’s image, and satisfying the yearnings and aspirations of the group and its members. Despite the competitive nature of the interactions of bees in the hive and the power stratification, cooperation nonetheless produces honey. Similarly, human communities can foster cooperation when they work together to achieve common goals.

A duty of coagency derives from the virtue of cooperation. Coagency means remaining responsible to one another and trusting that individual and collective actions and inactions are essential in making each other personhood. In sub-Saharan cultures, a dominant anthropological conception of being holds that “a person is a person through other persons or I am because we are” [38], often summarised as *ubuntu*. A relational phrase of having one’s personhood connected to the other, both ontologically and dutifully, in Yorùbá thought is captured in proverb – (iv) “*Èèyàn loògùn èèyàn*” (the being and wellness of a person is connected to the being and wellness of other people) [36]. This finding suggests that, to the extent that one’s personhood is determined by how well one relates to the other within the community, each personhood has a duty of coagency in being responsive and responsible. In doing so, moral premiums are given to people who share special relationship(s) (in groups). However, given human vulnerability to changing and changeable existential conditions, moral agents also have duties to be coagents with others with whom they (may or may not) potentially share common or similar interests and experiences. Exhibiting solidarity with ‘others’ in this way implies having a coagency duty to people with no immediate special relationships (out-groups). A duty of coagency in in-group and out-group relationships is pressing, especially in existential conditions.

Sharing is another virtue in the beehive metaphoric conception of solidarity in Yorùbá relational culture. Sharing is a norm in the hive as the foragers do not keep all the nectar they fetch for themselves. All bees in the hive, regardless of their role, benefit from the hive’s resources. A Yorùbá proverb that expresses sharing as a driver of positive human relationships is (v) “*J*ẹ *kí nj*ẹ *ní *ḿ*mú ayò dùn* (sharing alike makes for a harmonious relationship) [36]. As a marker of solidarity, sharing is not merely about basic resources; it extends to nonmaterials such as emotions, knowledge, power, and other existential situations of conviviality and agony. However, sharing is not without its limits. Caution is advised when sharing unduly places one in the most vulnerable position of self-harm. Hence, the proverb is as follows: (vi) “*Oko kì í je ti baba àti t*ọ*m*ọ *kó má nìí àlà*” (regardless of sharing or owning some commonwealth together, parties may benefit from boundaries) [36]. The import of this proverb is that it urges sharing up to the point that someone does not harm themselves in supporting others. Although sharing does not necessarily entail caring, it may indirectly oblige a caring enactment when one considers the element of reciprocity in solidarity.

The duty of reciprocity results from sharing. Reciprocity is about having a caring, deferential disposition to the vulnerability and situatedness of the conditions of others, and it does not mean being symmetrically reciprocal in good/bad gestures. A proverb that underscores this duty of reciprocity with people with whom one shares certain relations is as follows: (vii) “Ẹ*ni tí ó gòkè*,* kó fa ore-re lowo; *ẹ*ni tó rí j*ẹ,* kó fún or*ẹ *re j*ẹ” (whoever has reached the top, let him or her pull a friend by the hand; whoever has privileges, let him or her share it with the underprivileged) [36]. Reciprocity holds a minimalist duty to share, especially with the weaker party in solidarity relationships. The presumption here is that in solidarity relationships with others, there is an asymmetry of exchanges and cost-sharing expected by the solidaristic parties. Amongst the Yorùbá is the popular saying that (viii) “ṣ*e-fún-mi-kí-n*ṣ*e-fún-*ọ *loògùn ore*” (literally translated, this means “you-do-me-a-favour-and-I-do-you-a-favour is the medicine for friendship”) [36]. No matter how minimal the cost-sharing may be, solidarity is of less or no instrumental value for harmony when solidarity parties are empty-handed. For this reason, a Yorùbá proverb holds that (ix) “*Àj*ọ*j*ẹ *ò dùn bí *ẹ*nìkan ò ní; bí a bá ní là ń*ṣ*e àj*ọ*j*ẹ” (sharing is not pleasant if one party has nothing; sharing involves an expectance of something in return, no matter how disproportionate) [36]. Unlike some understandings of solidarity in Western culture, which entail standing with and for those who cannot help themselves without anything expected in return [12, 39, 40], an African conception of solidarity accepts standing in the gap for the weak within in/out-groups with some reciprocity in return, regardless of how disproportionate such solidaristic gestures might be.

Participation is another virtue to exhibit in the form of solidarity. Participation is about taking part in affairs that impact one, including being involved in their decision-making. Participation is a norm that keeps the hive as a functional unit of cohesion. To protect (and create) the colony, each bee becomes involved and partakes in the colony’s activities according to its capacity in a naturally assigned role. Given the distribution of roles among bees in the hive, the virtue of participation allows each bee to trust the other in discharging specific responsibilities to meet the hive’s needs. Consider swarming, for instance, where bees participate and decide together in finding and settling in a new hive. Amongst the Yorùbá, the virtue of participation is expressed in the proverb: (x) “*bí ojú bá ko ojú*,* àlà yó t*ọọ *nídìí ìgbá*” (when all parties participate in dividing something among them, no one is cheated)” [36]. This proverb speaks not only to being present at the table when a decision about what might affect one’s affair is being taken but also, figuratively, having a ‘voice’ and ‘hand’ on the matter to allow fairness in the process and outcome.

Reflexivity is a duty that flows from developing a virtue of participation as a way of life. A duty to be reflexive in a solidaristic relationship with others points to self-conscious reflection and appraisal of one’s influences, positions, and power in participation. Members of solidaristic relationships have a duty to continuously examine their attitudes, biases, privileges, and even prejudices to show empathy and support others within the group. A Yorùbá proverb emphatic on self-awareness as an important duty to exhibit is (xi) “*Ìpàko onípàko là ńrí; eni*ẹ*l*ẹ *ni ní ńrí t*ẹ*ni”* (one sees only the back of other people’s heads; only others can see one’s own) [36]. This proverb urges self-awareness and admonishes that one learns to pay closer attention to another’s situation rather than gazing at other people’s faults. Until this responsibility of self-awareness is taken seriously, the narrative of a solidary relationship will continue gazing at others.

Hospitality is also an important virtue of solidarity from a beehive metaphor with an African interpretation. Being hospitable is to be kind, generous, welcoming, and caring not only for guests but also for strangers. The prioritisation of the survival of vulnerable larvae and pupae highlights the moral importance of care during the hive. The close-knit dependency of bees on one another in the hive and the bringing and welcoming of nectar to the hive may be analogous in terms of hospitality. In Yorùbá culture, hospitality is a well-recognised virtue that places responsibility on the part of the host and the guest, as each is expected to be hospitable to each other. This point is emphasised in the proverb: (xii) “*Ojúlé ló bá wá; ebùrú ló gbà lo; ó dÍfá fún àlejò tí ńfe obìnrin onílé*” (whoever abuses hospitality will depart in disgrace) [36].

The duty of the accommodation of out-group members by in-group members is derivable from the virtue of hospitality. Accommodation is the recognition of differences and respect for others, even where aligning diverging interests among solidistic groups is impossible. A duty of accommodativeness presupposes conflicting and nonconflicting difference(s) between parties, which further requires some selflessness and/or sacrifice. The drones, for example, sacrifice their lives for survival and good of the hive by leaving the hive, especially during autumn, when foraging becomes more challenging, to allow the other bees to have enough honey produced at lower cold temperatures to feed on. In the human relational context, the Yorùbá appreciate sacrifices but are also cautious about the limits of sacrifices. Making sacrifices should not mean serving as a sacrificial lamb; instead, sacrifices mean give-and-take, letting go, or accepting to carry costs up to a point for the good of the other. Hence, the proverb (xiii) “*M*ọ *ìwà fóníwà loògùn ore”* [36], which means that knowing and tolerating each person’s character differences, is indispensable to a friendly relationship.

The point in the preceding discussion is that the beehive is a metaphoric symbol of solidarity in Yorùbá thought, and solidarity has some virtues analogous to those of the beehive. Solidarity, in this sense, refers to continuous inclination and attitudinal commitment towards building positive relationships with the *other*, which can be proximately or distantly situated within or across borders. When asked, ‘Why are in solidarity with the *other*?’, a typical response supported by the Yorùbá oral tradition is that “it is for the sake of harmony.” Harmony, understood as ‘ibaarepo’ in the Yorùbá language, is an experiential state of “enjoying a sense of togetherness” [41] for its own sake and not instrumentally. It is an ultimate value in the Yorùbá culture, as evidenced in the Yorùbá divination system, *Ifa*. *Ifa* is a literary corpus, an intangible cultural heritage, which archives “Yorùbá history, philosophy, medicine and mythology [and religion]” [42]. Harmony is an intrinsic good, and it is achieved through solidarity. This point is well emphasised in the *Odù *Ọ̀*sá’gúndá* verse of *Ifa*:

All goodness became a grouping together in harmony.

The grouping together of strands of hair covered the head….

The grouping together of trees became a forest….

Brooms are formed from bundles of twigs….

Beehives form swarms.

It is as swarms that the locusts consume the farm.

It is in several colonies that we find the termites in their mounds.

It is in groups that we encounter dragonflies….

So that the goodness of togetherness could come forth at once.

Indeed, all goodness took the form of a gathering together in harmony… [43].

The above *Ifa* narrative makes a normative conclusion about the intrinsic good of harmony from the premises consisting of natural observations. The metaphor is not about an individual honeybee in itself but about the hive, the capacity for swarming, which is phenomenally coming together with bees in solidarity. The instinctive behaviours of bees, social organisation, resilience, and symbolic communication of the hive are fascinating metaphors for staying in solidarity and keeping up struggles for improving social order. However (again) the paper does not claim that the beehive is the perfect analogy. The hive metaphorically represents solidarity, and honey is symbolic of the good of harmony. While solidarity is instrumental value, harmony has intrinsic value. Harmony is a disposition to act in ways that advance the balancing of discordant interests in social and heterogeneous relationships. It is “the search for equilibrium in social and political life” [44]. Thus, within Yorùbá culture, solidarity is central to achieving harmony. A lack of harmony creates discord, reinforces injustices, and eliminates the realisation of the common good. When the virtues of cooperation, humility, sharing, participation, and hospitality, which apply to in-and-out groups, are cultivated and put into practice by their corresponding duties of coagency, listening, reciprocity, reflexivity, and accommodation (Fig. 1), then solidarity is in action, and harmony can be realised.

## Potential objections and counterresponses

This section considers possible objections and responses to the above decolonial interpretation of the beehive metaphor as solidarity. First, reiteratively, a case for an African (decolonial) conception of solidarity is not a claim that the beehive metaphor is a perfect allegory for understanding solidarity.

First, critics might argue that anthropomorphising the bees’ observable characteristics as a metaphoric symbol of solidarity is taken too far and unacceptable. A counterresponse to this charge is that although bees have superorganismic features, a metaphoric analysis of their behaviours in anthropomorphic terms is valid because of the capacity of humans to imaginatively reflect on their humanity in light of their collective observations and discussions about the natural world. A consideration of the beehive metaphor attempted in this article is not a superfluous anthropomorphising of the bees’ behaviours but a cognitive way of doing ethics in an African traditional context through the use of folklore, metaphors, and proverbs in teasing out analogous virtues for human societies. It is a valid decolonial approach to thinking, generating knowledge, and unpacking complex ideas. The beehive metaphor is used here as a reflective method to develop a conception of solidarity. To that extent, the decoloniality of solidarity proposed in this article is at both the conceptual and the methodological levels. The beehive metaphor used as the cornerstone of this article’s conception of solidarity is not literally oversimplified but thoughtfully and analogously reflected in the analysis of solidarity using African proverbs as ways of knowing.

Second, framing solidarity as both a virtue approach and a deontological approach to morality with derivative duties appears contradictory. Accepting a deontological dimension within the virtue-ethical approach is not problematic within the logic that underpins African epistemologies, as “virtue development is essential to carry out obligations” [33] and vice versa. As opposed to a logic of “either …or…” (which is a bivalent logic; a logic of exclusion) that characterises some Western intellectual traditions, the Yorùbá conceptual scheme is premised on a logic of inclusion (that is, complementary or trivalent logic) where contradictions in thought are value-complementarity [45, 46]. Accordingly, this logic informs an African conception of solidarity, and it takes the virtue dimension of solidarity as primary and derivative duties as complementary and, therefore, not contradictory to virtues. In line with this logic, other African conceptions of solidarity identify its duties [21, 22].

Third, critics might query the seeming caste system of the beehive into worker bees, queens, and drones and contend that the idea of the choice of roles and duties is absent within the biologically determined roles of the bees. This objection is a significant drawback for a conception of solidarity because it might reinforce power hierarchies and ethical imperialism. Rather than attenuating the plausibility of the beehive metaphor as an aspirational ideal for solidarity on the grounds of a lack of individuality, choice, and control, it arguably embeds these elements within it. The alleged ‘hierarchy’ of a beehive ‘caste’ system is an anthropomorphism. Beehives have no hierarchy. The queen is not a queen in any meaningful sense, which applies to human societies. She is more of an egg-laying machine. Instead, what is clear is that each serves a purpose in the hive for the flourishing of the hive, a purpose not enforced by some oppressive structure but rather by a natural process in the hive. Thus, the exercise of choice and control is a function of the depth of character development a moral agent can nurture within a social space. As natural as the hierarchies and powers in human societies may be, or products of artificial creation as they might appear, their impacts are minimisable through continuous struggle for choice and control by moral agents.

Fourth, another possible objection is that an African conception of solidarity does not deserve to be taken seriously. Given the sociopolitical adversities that historically plagued Yorùbá culture, solidarity and its virtues are mere rhetoric that do not translate into real lived-world experiences in such a culture. In reality, however, solidaristic practices are operationalised in many spheres of life in traditional Yoruba societies. Though abuses of solidaristic encounters do occur, they are not unique to the Yorùbá, and they suggest that the value of solidarity is not an expression of a perfect state of moral existence. Instead, it is an ideal worth striving for as a metaphorically constructed excellence in Yoruba (nay African) culture(s); imperfect character and relationships require continuous efforts at pursuing moral excellence. Dismissing an African conception of solidarity as mere rhetoric and ignoring its potential to enrich global health research practices risk perpetuating epistemic injustice and delegitimising an ethical framework central to and dominant in many African communities. Such a conception of solidarity fosters moral responsibilities and duties to the similarities of vulnerabilities across borders, emphasising mutual interdependence and equitable relations, which are core to global health and ethics. Besides the extant conceptions in Western literature, other voices on solidarity deserve to be heard, too, as a matter of epistemic justice. An African conception of solidarity can complement extant conceptions of solidarity in expanding the decolonial moral-epistemic gaze in global health ethics.

Fifth, critics might raise further objections that an account of solidarity articulated in this paper is not necessary for decoloniality, given the extant African accounts of solidarity in philosophy and global health ethics literature. While there are other accounts of solidarity within the African philosophical space [18–20, 34, 46–50], this article adds to the extant discourse on solidarity by employing the decolonial methodology of the palaver and metaphor common to many African societies in constructing a normative account of solidarity. Other African concepts of solidarity are not derived via such methods. In itself, the methodology of palaver and metaphor challenges the epistemic colonisation of modes of interpretation and can be utilised beyond this paper when values, concepts, and relations of norms in global health ethics are explored. This decolonial methodology can be helpful in advancing epistemic justice and expanding the discourse on solidarity and other concepts within the subfield.

The African account of solidarity articulated in this article employs proverbs, metaphors, and parables that resonate with and beyond the African people. While such an account of solidarity is valid from an African perspective, it may thus also resonate with many non-African intellectual cultures. Accepting such a metaphor enables the possibility of engaging the minds and imaginations of other societies familiar with how bees operate and what the beehive metaphor might represent differently. By articulating the integrative virtues and duties of solidarity derived from the beehive metaphor, which are gaps in the extant African literature [18–20, 34, 46–50], this article provides a novel contribution to the decolonial discourse on solidarity in bioethics and philosophy.

The following section exemplifies how the preceding conception of solidarity applies to global health research.

## An african conception of solidarity and funding global health research

Deeply entrenched asymmetries in power and privileges in global health research give rise to disparities in who benefits most from research and who gets research funding. Funding institutions in high-income countries (HICs) control resources and, therefore, can determine research priorities [51, 52]. “When HIC donors give grants, they predominantly fund institutions, contractors, and principal investigators in their own countries. For example, 70% of Fogarty grants go to US and HICs, 73% of Wellcome Trust grants support UK-based activity, 80% of USAID contracts go to US firms, and 88% of grants by the BMGF is estimated to be held by global North institutions” [52].

An African conception of solidarity derived from the beehive metaphor holds promise in challenging and ultimately reforming the asymmetries that give rise to these unjust disparities. First, it discourages differentiated othering and affirms a single community. Second, the virtue of humility and the duty of listening encourage dialogue and consensus-building as the primary mode of setting global health research priorities. Third, through the virtue of sharing and duty of reciprocity, it provides the moral justification for proportionately pooling global resources. Fourth, the virtue of cooperation and the duty of co-agency can facilitate more equitable research into often neglected diseases and the involvement of those most affected. Fifth, the virtue of participation and its duty of reflexivity can ensure that all are included and power imbalances are continually corrected.

First, differentiated othering is when differences are used to undermine the interconnectedness and similarities among groups and individuals. In the beehive metaphor, the queen, just like the drone or the workers, does her job without prejudice or bias against others (drone and workers) in the hive. There is no identity conflict in the hive where one category of bees undermines the other because of pecuniary interests, hierarchical positioning, or primordial identities. Rather, all bees form an interdependent community that thrives because of individual differences. Similarly, in reforming the global health funding system, all stakeholders must play their roles to sustain the global community rather than engage in differentiated othering to support their own interests. When stakeholders see and act on the precept of an interconnected and interdependent world as more fundamental, differences become the basis for cooperation towards equitable institutional reforms in the global health research funding ecosystem [53].

Second, recognising the solidarity of a single community gives rise to the virtue of humility and the duty to listen to others since the community must thrive. Through active listening and commitment towards being heard, dialogue and consensus-building can be enabled amongst stakeholders in global health funding. Global health reform can be achieved through dialogue and consensus-building, which is integral to the palaver approach used in deriving an African conception of solidarity. Centering humility in political reforms encourages participation and power negotiations across stakeholder divides. In so doing, the tendency towards epistemic arrogance among HICs on what constitutes health priorities amongst disadvantaged research communities will be ameliorated through recognition of the disadvantaged communities’ epistemic capacity as equal partners with valuable contributions to make to the research project. Humility necessitates a duty to listen, which requires learning from the lived experiences of marginalised/disadvantaged communities on the most pertinent health needs that can shape the content of health research priorities and funding calls. Through embedding the solidarity virtue of humility and its derivative duty to listen, LMIC researchers and the marginalised/disadvantaged communities should have a voice and an influence in shaping global health research funding priorities with more equitable outcomes that meet the disadvantaged people’s needs than what the current aid-driven funding system provides.

Third, guided by the virtues of sharing, global health research funding can be sourced through a global pooling of human and non-human resources amongst partners. The duty of reciprocity derived from the virtue of sharing in an African conception of solidarity means that some level of resource contributions to the worldwide pool is expected from LMICs, at least in proportion to their resource endowments and capabilities. A sharing relationship between actors within and across HICs and LMICs is one without judgments of inferiority on the amount of financial resources that partners from LMICs bring to the pool. In such financial reform initiatives, funding organisations will primarily serve as solidarity builders and trustees of financial and non-financial resources proportionately pooled by all stakeholders. When global health funders become solidarity builders, they are trustees of the collective resources and are expected to act in solidarity with other stakeholders, who have shared powers, voices, and influences on the governance of global health research funds. Just as bees in the hive, regardless of their status and role, equitably benefit from the hive’s nectar, global health research funders as solidarity builders will be responsible for enabling sharing through funding research that advances health equity.

Fourth, the virtue of cooperation in an African conception of solidarity leads to a duty of co-agency in decisions on how resources are allocated, which will lead to better funding of effective research on neglected diseases such as malaria or tuberculosis that largely affect marginalised and disadvantaged populations in places like Africa. Cooperation presupposes interdependence. The shared human vulnerability to diseases, whether emerging infectious or neglected tropical diseases, underscores a similarity of identity. Just as all bees in a hive are vulnerable to disease, all humans are vulnerable to diseases, especially in a globalised world that forms a single community. Such relevant similarity should encourage a cooperative response to disease risk.

The duty of co-agency in global health research funding entails having shared ownership and joint accountability. Where there is a duty of co-agency, all stakeholders are equal collaborators regardless of geographical provenance or resource standing in the global health research pool of resources. Co-agency in global health research funding practices will mean that funding institutions must involve stakeholders from marginalised communities across the projected research population in co-designing research funding calls with substantive representation and power on the board that decides which research protocol to fund. The power dynamics of global health funding institutions can shift from hierarchical to collaborative through cooperation and the duty of co-agency. Incorporating co-agency is necessary for linking global health research funding to reducing global health disparities. To illustrate, in meeting the pressing health needs of marginalised poor populations through global health research impact fund interventions, a duty of co-agency can translate into allocating resources for neglected-disease research that disproportionately impacts low-resource settings. Funding such research through the pooled resources from HICs and LMICs is justified based on the similarity of needs and not profitability, affordability, and accessibility with high impact over patency.

Fifth, the virtue of participation and its corresponding duty of reflexivity is required in such global pooling of funds to ensure effective participation of all sovereign states with reflexivity as a means of balancing power plays in the sharing relationships. Reflexivity is continuously required in solidaristic relationships as a self-corrective duty owed by global North and global South stakeholders in evaluating possible biases, privileges, and power dynamics in their actions and impacts of their interventions. In the context of global health research, funders have reflexive duties to continuously reform and resist invisible structures that foster inequities in health outcomes through conscious commitments to fair power and resource sharing. Upholding a duty of reflexivity calls for re-examining extant practices of aligning resources with external agendas to support research protocols that genuinely serve the interests of the solidarity party identified with relevant similarity of need(s). Beyond the funders, other stakeholders, including researchers and communities, also have an ongoing duty of reflexivity on their actions as agents of solidarity and how such impact global health research equitable partnerships.

Funders must reflect on whether their funding structures perpetuate power imbalances by favoring high-income country researchers or projects detached from the realities of the host communities. Reflexivity calls for recalibrating funding criteria to prioritize initiatives co-designed with local stakeholders, ensuring resources address locally identified needs rather than external agendas. This might involve shifting from short-term, output-focused funding models to long-term investments that build sustainable local capacities, much like the beehive’s reliance on collective effort to sustain its future.

The recommendation on pooling of resources for global health research derived from an African conception of solidarity based on an interpretation of a beehive metaphor is similar, in practical terms, to past proposals such as the 2011 proposal on “Essential Health and Biomedical Research and Development Treaty” [54] by Health Action International Global (HAIG), a joint NGO initiative for Health and Equity in Society, submitted to the WHO Consultative Expert Working Group on Research and Development: Financing and Coordination, Thomas Pogge’s 2012 proposal on the “Health Impact Fund” (HIF) [55], and a recent 2023 proposal by Chilufya et al. on “The Ubuntu Health Impact Fund” [56]. These related proposals are important first steps in complementarily transitioning from the current global health research funding sources from donors and international institutions in HICS with patency as innovation reward and incentives for investment in pharmaceutical research and development to a funding model that obligates national government and international institutions to equitable sharing of the costs, access, and benefits of research and development in ways that enable innovators to get rewarded according to the social benefit and impacts achieved through their innovation made available at a non-profit price in resource-poor settings. While the 2011 R&D treaty proposal is designed within a human rights prism, and the (Ubuntu)Health Impact Fund is framed within global health justice, an African conception of solidarity offers relevant and complementary foundational virtues and duties that support the pooling of funding and equitable sharing of research benefits that the proposals also suggest. An African conception of solidarity supports initiatives such as HIF that obliges global institutions and sovereign states to contribute towards advancing health equity not as charity but as a matter of moral obligation [57].

## Conclusion

This article decolonises solidarity in global health ethics by unpacking an African conception of it using a beehive metaphor. A beehive is a metaphoric symbol of solidarity, expressing social relations in which people stand together (stand with and stand for) for collective action. Humility, cooperation, sharing, participation, and hospitality are salient virtues derived from the beehive metaphor. Using traditional Yorùbá approaches that use metaphors, proverbs, and interpretations, the identified virtues have corresponding relational duties of listening, coagency, reciprocity, reflexivity, and accommodation, respectively.

In challenging the subordination and erasure of methods of discovering ethics norms from the Global South, this article employed a metaphoric approach to knowledge production as an instance of methodological decoloniality. An African conception of solidarity is unpacked in this article with an example of what it means for global health research funding. Such a conception can serve as a starting point for further conversations with other extant perspectives on solidarity from the Global South and the Global North. An allegorical interpretation of a beehive metaphor provided can enable further inter- and transcultural conversations with other possible related metaphors for solidarity. As akin to the palaver as a space for hearing the voices of every epistemic agent, this article invites readers to reflect on solidarity in global health ethics through metaphor and heuristic interpretations in a community of dialogue.

Scholars are, therefore, invited to enter into the spirit of metaphorical language, proverb and communal interpretation by responding to an African conception of solidarity articulated in this article or extending it. For example, in the spirit of a palaver, investigating the nuances of harmony and whether it can serve as an overarching guiding value in global health ethics might be interesting. While this article is perhaps limited in attending to this question, another related fundamental question that this article does not directly consider but may generate interest for future studies in global health ethics is as follows: what other kinds of structures or restructuring are needed in operationalising and advancing conceptions of solidarity within the emerging global health research decoloniality space? What kinds of trade-offs, freedom, for example, would a solidarity regime of ethic spark in global health research decoloniality, and what ordering principles should come to play?

## Data Availability

No datasets were generated or analysed during the current study.

## Footnotes

1 While recognising that the binary of Global North and Global South is under contestation particularly with regard to whether states occupying an interstitial position between North and South deserve the label of global East [16], this paper adopts the popular geopolitics of knowledge and power classification. The understanding of the term global North within this paper is a postcolonial and epistemological construct broadly classifying knowledge production from the universalism in the geopolitical North with more political and economic powers. The Global South is taken to mean both postcolonial and epistemological classification of the multiplicity of knowing practices in the Global South with majority population but lesser political and economic powers in global affairs.

2 The Yorùbá are largely located in Southwestern part of Nigeria though widely spread in many parts of the country, in West Africa and other parts of the world. The choice of Yorùbá culture amongst the multiple ethnic groups in South Sahara is a matter of convenience in understanding their linguistic resources for heuristic analysis.

3 The proverbs elicited in this article are in Roman numerals in ascending order for the sake of organisation and logical discourse flow in relation to the virtue or duty under reference.
